# Zika virus infection of the placenta alters extracellular matrix proteome

**DOI:** 10.1007/s10735-021-09994-w

**Published:** 2021-07-15

**Authors:** Gabriel Borges-Vélez, Julio Rosado-Philippi, Yadira M. Cantres-Rosario, Kelvin Carrasquillo-Carrion, Abiel Roche-Lima, Jean Pérez-Vargas, Andrea González-Martínez, María S. Correa-Rivas, Loyda M. Meléndez

**Affiliations:** 1 Department of Microbiology and Medical Zoology, School of Medicine, University of Puerto Rico Medical Sciences Campus, San Juan, PR, USA; 2 Department of Biology, University of Puerto Rico Rio Piedras Campus, San Juan, PR, USA; 3 University of Puerto Rico Comprehensive Cancer Center, San Juan, PR, USA; 4 Center for Collaborative Research in Health Disparities (CCHRD), Research Infrastructure Core, University of Puerto Rico Medical Sciences Campus, San Juan, PR, USA; 5 Department of Pathology and Laboratory Medicine, University of Puerto Rico School of Medicine, San Juan, PR, USA

**Keywords:** Zika virus (ZIKV), Tandem mass tagging (TMT), Ingenuity pathway analysis (IPA), Extracellular matrix (ECM), Fibrinogen (FG), Congenital zika syndrome (CZS)

## Abstract

Zika virus (ZIKV) infection has been associated with fetal abnormalities by compromising placental integrity, but the mechanisms by which this occurs are unknown. Flavivirus can deregulate the host proteome, especially extracellular matrix (ECM) proteins. We hypothesize that a deregulation of specific ECM proteins by ZIKV, affects placental integrity. Using twelve different placental samples collected during the 2016 ZIKV Puerto Rico epidemic, we compared the proteome of five ZIKV infected samples with four uninfected controls followed by validation of most significant proteins by immunohistochemistry. Quantitative proteomics was performed using tandem mass tag TMT10plex™ Isobaric Label Reagent Set followed by Q Exactive™ Hybrid Quadrupole Orbitrap Mass Spectrometry. Identification of proteins was performed using Proteome Discoverer 2.1. Proteins were compared based on the fold change and *p* value using Limma software. Significant proteins pathways were analyzed using Ingenuity Pathway (IPA). TMT analysis showed that ZIKV infected placentas had 94 reviewed differentially abundant proteins, 32 more abundant, and 62 less abundant. IPA analysis results indicate that 45 of the deregulated proteins are cellular components of the ECM and 16 play a role in its structure and organization. Among the most significant proteins in ZIKV positive placenta were fibronectin, bone marrow proteoglycan, and fibrinogen. Of these, fibrinogen was further validated by immunohistochemistry in 12 additional placenta samples and found significantly increased in ZIKV infected placentas. The upregulation of this protein in the placental tissue suggests that ZIKV infection is promoting the coagulation of placental tissue and restructuration of ECM potentially affecting the integrity of the tissue and facilitating dissemination of the virus from mother to the fetus.

## Introduction

Zika virus (ZIKV) belongs to the family Flaviviridae, genus Flavivirus, considered an arbovirus as a result of being transmitted mainly through insect bites. It is transmitted in the urban environment by Aedes mosquitoes, which also transmit other arboviruses like dengue virus, chikungunya, and yellow fever virus. This virus was first isolated in 1947 from a sentinel rhesus monkey (*Macaca mulatta*) in the forest of Zika in Uganda Africa (Dick et al. 1952). However, ZIKV emerged for the first time in the Americas including Puerto Rico in 2015–2017 with 738,783 infected people and inducing perinatal disease that affected 2635 children (CDC 2016). In recent years, the number of infections decreased. By 2020 only 43 symptomatic cases have been reported so far by the CDC in the US and territories (CDC 2020). Described as a Dengue-like disease with no significant health complications when originally discovered, ZIKV mutated to produce a disease with significant health complications including Guillain-Barré Syndrome (GBS) (Parra et al. 2016). Although up to 80% of ZIKV infections are asymptomatic, this infection can produce the following symptoms: conjunctivitis, skin rash, high temperature, and joint pain. Sporadic sexual and perinatal transmission have been described, as well as transmission from blood and platelet transfusions. Transmission of ZIKV evolved to a sexual and vertical mode of transmission, being the first flavivirus to acquire these characteristics (Foy et al. 2011).

ZIKV infection is characterized as a self-limiting disease; however, during pregnancy, vertical transmission is associated with fetal abnormalities such as microcephaly, brain microcalcification, central nervous system disorders, ocular abnormalities, and other manifestations (Moore et al. 2017). The aftermath of the infection during pregnancy is known as congenital zika syndrome (CZS). Common placental tissue alterations include delayed villous maturation and additional stromal changes such as Hofbauer cell (HC) hyperplasia (Roseneberg et al. 2017). ZIKV can infect different placental cell types, including the two cells that confer immune protection against pathogens: Hofbauer cells (HC) or placental macrophages and trophoblasts (Aagaard et al. 2016; El Costa et al. 2016; Tabata et al. 2016; Jurado et al. 2016; Quicke et al. 2016; Simoni et al. 2017; Noronha et al. 2018). Trophoblasts are the major constituent of the placenta forming a continuous physical barrier, which could explain some of the reported abnormalities caused by ZIKV in the placenta. Nonetheless, it is unknown why vertical transmission does not occur in all pregnant women infected with ZIKV and symptomatic congenital infection is not observed in all exposed fetuses. The exact mechanisms used by ZIKV to interfere with the placental barrier are still unknown, leading us to investigate the proteome of the placenta from ZIKV infected women using a quantitative proteomics approach. Flaviviruses can affect extracellular matrix (ECM) proteins (Folly et al. 2011; Tomlin et al. 2018; Besson et al. 2020; Aguiar et al. 2020), thus we hypothesize that ZIKV infection of placental tissue deregulates ECM proteins promoting the loss of placental integrity, persistent infection, and transplacental transmission. To test our hypothesis, we analyzed the proteome of placenta samples from nine Puerto Rican ZIKV infected women collected during the 2016 pandemic and compared them to four uninfected controls by tandem mass tagging (TMT) quantitative proteomics.

## Materials and methods

### Ethics approval

This study is currently approved by the Institutional Review Board (IRB) of the University of Puerto Rico Medical Sciences Campus (UPR-MSC), Protocol Number 0720119, entitled Role of placenta cystatin B in mother to child transmission of Zika virus. The principal investigator of the protocol is Dr. Loyda M. Meléndez. This protocol did not involve direct interaction with human subjects. We did not perform recruitment, because the 21 placenta samples were provided by Dr. Maria Correa from the UPR-MSC Department of Pathology and given to us with no patient identifier. Placentas were collected with approval from subjects of legal age, 21 years or more. Information provided to us included gestational age, mode of delivery, and if Zika virus infection occurred during gestation. With this information we divided our samples into two groups; positive and negative for Zika infection. No other information about the placental samples was available to us, and no knowledge if congenital zika syndrome (CZS) was an outcome. No staff member or student have any financial interest such as royalty or any other payments in sponsor or other entities having a financial interest in intellectual property, product, or services. The research was performed complying with all the University of Puerto Rico Medical Sciences Campus policies and procedures, as well as with all applicable Federal and State laws regarding the protection of human participants in research, protecting the rights and welfare of human participants, the conduct of the study, and the ethical performance of the project.

### Consent to participate

Consent to participate was not performed because the placenta samples were taken from processed/examined placentas at the Puerto Rico Medical Center and were given to us without patient identifier. The Institutional Review Board (IRB) of the UPR-MSC declared that our protocol was exempt from the consent of participation, category 4 because the placenta samples were given to us without patient identifier.

### Patient selection and specimens

Placenta samples were collected from already processed/examined placentas stored at the Laboratory of Anatomic Pathology, ASEM, Puerto Rico Medical Center, and paraffin blocks were prepared and labeled with a unique number but no patient identifiers. Zika infection during pregnancy of these samples was determined by RT-PCR performed at the Puerto Rico Department of Health and only the results were available for this study. Placentas were grossly examined and microscopic sections evaluated for pathologic abnormalities by Dr. María Correa. Pathologic findings were not disclosed as part of the study because this is confidential information for the subjects and they were not followed-up after delivery. Samples were collected during the 2016 ZIKV epidemic and stored in 10% buffered formalin solution for preservation and fixation. In our study, we randomly chose placental tissue from five ZIKV infected and four negative control women available at the Pathology repository following Protocol Number 0720119, approved at the UPR-MSC. Sections of the preserved placental tissue containing chorionic villi was processed overnight and paraffin blocks 4 μm thick sections were cut using a microtome and mounted on charged glass slides by the histotechnologist at the Department of Pathology, UPR-RCM.

### Protein extraction from paraffinized tissue

Proteins were extracted from 3 slides of each placenta sample using the Qproteome FFPE Tissue Kit (20) (QIA-GEN; Hilden, Germany) following the manufacturer’s instructions. Slides were deparaffinized in Xylene for 10 min at room temperature, rehydrated by incubations in 100%, 96%, and 70% ethanol for 10 min at room temperature twice, followed by rehydration in ultra-pure water for 30 s. The whole tissue in the slide was removed with a needle and transferred to a 1.5 mL microtube containing 100 μL coExtraction Buffer EXB Plus supplemented with βmercaptoethanol. The samples were homogenized by vortex at RT and incubation in a heating block at 100 °C for 20 min followed by a Thermomixer at 80 °C for 2 h with agitation at 750 rpm. After heating, the tubes were incubated at 4 °C for 1 min and centrifuged at the same temperature for 15 min at 14,000× *g*. For protein precipitation, 1000 μL of cold acetone with 5% SDS was added to the supernatant and stored in the − 20 °C freezer overnight. Thereafter samples were centrifuged at 12,000× *g* at 4 °C for 15 min. The supernatant was discarded, the protein pellet was vacuum dried, and then resuspended in 100 μL of phosphate buffer containing protease inhibitors. Protein concentrations were determined using DC Protein Assay (Bio-Rad Laboratories; Hercules, CA, USA). Aliquots of 30 μg were vacuum dried and resuspended in 10 μL of SDS-PAGE sample buffer and loaded into precast polyacrylamide gel for electrophoresis (Bio-Rad Laboratories; Hercules, CA, USA), run at 95 V, and stained with Bio-safe Coomassie (Bio-Rad Laboratories; Hercules, CA, USA).

### Sample preparation for TMT-labeling

Protein bands in each lane of the Coomassie-stained gels, from nine samples containing 30 μg, were cut, diced into 1 mm cubes, distained (750 μL distaining solution of 50% acetonitrile and 50 mM ammonium bicarbonate) at 37 °C, reduced with 1,4-Dithiothreitol (25 mM DTT in 50 mM ammonium bicarbonate) at 55 °C for 30 min, and alkylated with 10 mM iodoacetamide in 50 mM ammonium bicarbonate for 45 min in the dark. Thereafter, samples were digested overnight at 37 °C using a 1:40 trypsin: protein ratio. For peptide extractions, gel pieces were first treated with a solution of 50% acetonitrile and 2.5% formic acid in water, followed by acetonitrile. Samples were subsequently dried and stored at − 80 °C before the TMT labeling procedure.

### TMT-labeling

Dried extracted samples were reconstituted in 100 mM triethylammonium buffer (TEAB) and labeled with TMT10-plex labeling reagents (41 μL, 0.8 mg) as specified by the manufacturer’s instructions (Thermo Fisher Scientific; Waltham, MA, USA). Following one hour incubation at room temperature, sample labeling was quenched with 5% hydroxylamine for 15 min. Equal sample amounts were pooled and dried in the Vacufuge Plus system (Eppendorf; Hamburg, Germany) for consequent LC–MS/MS analysis. The TMT pooled sample was further fractionated using the Pierce High pH Reversed-Phase Peptide Fractionation Kit (Thermo Fisher Scientific; Waltham, MA, USA) following the manufacturer’s instructions. Briefly, the column was conditioned twice using 300 μL of acetonitrile, centrifuged at 5000× *g* for 2 min, and the steps were repeated using 0.1% Trifluoroacetic acid (TFA). The samples were reconstituted in 300 μL of 0.1% TFA, loaded onto the column, and washed to remove salt contaminants or any unbound TMT reagent. The cleaned samples were then eluted 8 times with a series of elution solutions with different Acetonitrile/ Triethylamine (0.1%) percentages and subsequently dried before LC–MS/MS analysis.

### Sample preparation and LC–MS/MS analysis

Samples were reconstituted in 0.1% formic acid in water for Mass Spectrometry protein quantitation. For each sample, 2 μL were injected into the instrument, and the remaining volume was stored at − 80 °C. For peptide separation, the Easy-nLC 1200 HPLC system (Thermo Fisher Scientific; Waltham, MA, USA) was employed, and peptides were loaded onto a PicoChip H354 REPROSIL-Pur C18-AQ 3 μm 120 A (75 μm × 105 mm) chromatographic column (New Objective, MA, USA). The separation was obtained using 7–25% gradient Buffer B (0.1% formic acid in acetonitrile) for 102 min, 25–60% of Buffer B for 20 min, and 60–95% Buffer B for 6 min, for a total gradient time of 128 min at a flow rate of 300nL/min, and an injection volume of 2 μL per sample. Separated peptides were electrosprayed and analyzed using a Q-Exactive Plus mass spectrometer (Thermo Fisher Scientific, IL, USA), operated in positive polarity and data-dependent mode. The full scan/MS1 was measured over the range of 375–1400 at a resolution of 70,000, while the MS2 (MS/MS) analysis was configured to select the 10 most intense ions for fragmentation at a resolution of 35,000 with a dynamic exclusion parameter of 30.0 s.

### Database search

Following Mass Spectrometry analysis, raw data files were searched with a human database from UniProt (Universal Protein Resource, © 2002–2021 UniProt Consortium) updated in November 2017. Proteomics data was analyzed with Proteome Discover software version 2.1 using workflows configured for reporter ion quantification. The modifications included in the parameters were a dynamic modification for oxidation +15.995 Da (M), a static modification of +57.021 Da (C), and the modifications from the TMT reagents +229.163 Da (Any N Term, K).

### Statistical analysis and enrichment analysis of tandem mass tagging results

The bioinformatics analysis was performed by comparing the proteomic datasets from 5 replicates for Zika (+) group and 4 replicates for Zika (−) group (Table 1). The datasets were processed with custom python and R scripts before the statistical analysis for missing values, identification, and processing of replicates with outlier analysis using the Interquartile (IQR) mean imputation method. The statistical analysis was performed with the R-Bioconductor software *limma* (Ritchie et al. 2015). A single channel analysis was designed between each comparison of cases vs controls (Table 1). As a result, the list of proteins with significant differential abundance was obtained for each comparison, considering a fold change (FC) greater or equal to |2| (i.e. FC ≥|2|) and adjusted *p* value using the Benjamini–Hochberg method with a value less or equal to 0.05 (i.e. *p* value ≤ 0.05).

Ingenuity Pathway Analysis (IPA) was generated using their software (IPA®) The [networks, functional analyses, etc.] (QIAGEN Inc., https://www.qiagenbioinformatics.com/products/ingenuity-pathway-analysis) and used for enrichment pathway proteome analysis. The lists of proteins with significant differential abundance obtained from the statistical analysis were used as an input for IPA. IPA CORE analysis was applied in each case to identify (1) sub-cellular locations and protein annotations, (2) diseases and functions associated with a viral infection, (3) canonical pathways, (4) pathway predictions [using the Molecule Activity Predictor tools (MAP)], and (5) protein–protein interaction networks.

### Immunohistochemistry

Differentially expressed proteins and their location in the placenta were determined by 3-color immunofluorescence using 6 Zika positive and 6 Zika negative placenta, for a total of 12 samples. A total of 3 most significant proteins were selected for validation: fibronectin (FN), fibrinogen gamma (FGG), and alpha 1 antitrypsin (SERPINA1). Paraffinized embedded 4 μm tissue slides were deparaffinized placing them in Xylene for 30 min, three times. Rehydration of the tissue was achieved by submerging the slides for 3 min three times in serial dilutions of ethanol 100, 90, and 70% respectively. To completely rehydrate the tissue, slides were immersed in distilled water twice for 3 min. Antigen retrieval was attained by submerging the slides in a jar with boiling Citrate Buffer (10 mM, pH = 6) followed by cooling down for 30 min at RT and 30 min at 4 °C, completing the 60 min in citrate buffer. After the antigen retrieval, slides were washed with 1X phosphate buffer saline (PBS) three times while shaking for 5 min. Blocking was performed by adding a blocking solution consisting of 1% goat serum, 0.1% bovine serum albumin (BSA) in 1X PBS for 2 h, and constant shaking. Thereafter, the blocking solution was removed, and primary antibodies at optimal concentrations were added in combination with the blocking solution. Primary antibodies used were anti-Iba1 rabbit polyclonal antibody (FUJIFILM Wako Pure Chemical Corporation; Osaka, Japan) at 1:200 concentration to stain the HC and anti-CK8+18 Guinea Pig Polyclonal Antibody (Fitzgerald; Concord, MA, USA) at 1:200 concentration to stain the trophoblasts. For the validation of the proteins of interest, we used the mouse primary antibodies in separate slides with the stain for HC and trophoblasts, anti-fibronectin antibody [IST-9] (Abcam; Cambridge, United Kingdom) at 1:200 dilution to stain fibronectin, anti-alpha 1 antitrypsin antibody [B9] (Abcam; Cambridge, United Kingdom) at 1:200 dilution to stain alpha 1 antitrypsin, and anti-fibrinogen antibody (Abcam; Cambridge, United Kingdom) at 1:200 dilution to stain fibrinogen gamma. Slides containing the primary antibody and blocking solution mix were incubated by shaking at room temperature overnight. To remove the primary antibody, slides were rinsed with 1X phosphate buffer saline (PBS) three times while shaking for 5 min followed by secondary antibodies mixed at 1:200 dilution in blocking solution for 2 h in the dark. Secondary antibodies used were goat anti-mouse Alexa Fluor™ 488, goat anti-rabbit Alexa Fluor™ 546, and goat anti-guinea pig Alexa Fluor™ 647, all IgG (H+L) (Thermo Fisher Scientific; Waltham, MA, USA). The slides were rinsed with 1X phosphate buffer saline (PBS) three times while shaking for 5 min to remove the secondary antibody mix. After using a Kimwipe to remove any liquid residue, VECTASHIELD antifade mounting medium was added with DAPI for nuclear staining (Vector Laboratories, Inc.; Burlingame, CA, USA) and mounted with a coverslip. Slides were incubated in the dark at 4 °C for 24 h until the mounting media hardened, and nail polish was applied to the sides of the coverslips until dry.

### Immunofluorescence microscopy

Fluorescent labeled slides were examined using the Eclipse E400 microscope (Nikon Inc.; Melville, NY, USA). Pictures were taken with DS-Qi2 Monochrome Camera (Nikon Inc.; Melville, NY, USA) and the computer Imaging Software NIS-Elements A (Nikon Inc.; Melville, NY, USA). A total of five pictures of aleatory picked fluorescently labeled areas at a magnification of 20X were captured per slide. Aleatory areas of tissue were selected to capture the pictures based on similar cell ratios as determined by the number of nuclei. A total of four pictures were captured with different filters in the same area, one for each fluorescent label secondary antibody and DAPI using one second exposure. The wavelengths to excite each fluorophore were 358, 488, and 546, and 647 nm. Using the NIS-Elements A software tools of analysis, data were quantified from each picture by counting objects and mean fluorescence intensity of each labeled protein. Data was exported to the software Excel from Office 365® (Microsoft®; Redmond, WA, USA).

### Statistical analysis of immunofluorescence results

The Graph Pad Prism Software Inc. version 8.0.1 (San Diego, CA, USA) program was used for statistical analysis. Before any comparison, the data from the number of nuclei was submitted to a ROUT outlier test with *Q* = 1%. Samples that resulted in being an outlier for the number of nuclei were not considered for further comparisons. We tested for normal distribution by the Shapiro–Wilk test with a significance level alpha of *p* < 0.05 due to the small sample size. An unpaired t-test with Welch’s correction was applied using a 95% confidence as statistically significant (*p ≤ 0.05, **p ≤ 0.01, ***p ≤ 0.001, ****p ≤ 0.0001). The immunofluorescence data analyzed was considered semi-quantitative since is derived from pictures. Statistical comparisons were made between uninfected and ZIKV infected placentas for the number of nuclei, mean intensity of the labeled proteins of interest and mean intensity of labeled IBA1 to determine differences in the number of macrophages during Zika infection of the placenta.

## Results

### Differentially expressed proteins between zika positive and zika negative placentas by tandem mass tagging

**Fig. 1:**
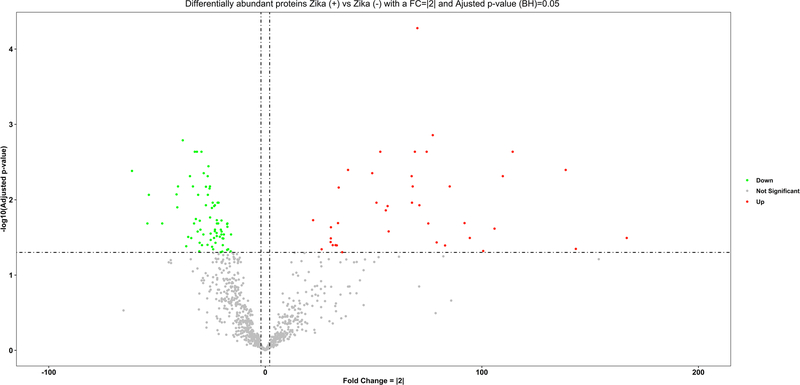
Volcano plot of differentigy abundant proteins in Zika (+) versus Zika (−) placenta. Data is stratified as More abundant (red dots) or Less abundant (green dots) using the criteria of FC >|2| and a *p* value adjusted with the Benjamini–Hochberg method of ≤ 0.05. (Color figure online)

We successfully isolated proteins of preserved paraffinized placental tissue from Zika positive mothers and uninfected controls. The maximum number of proteins identified from a sample was 1297. The efficiency of the peptide TMT labeling was 96%, indicating that the protocol was successful and that appropriate quantitative comparisons between proteins could be performed. A total of 113 proteins were significantly differentially expressed in ZIKV positive placental tissue (Supplementary Table 1). The volcano plot shows the proteins arranged by their increased or decreased fold change and their significance. The more abundant proteins can be observed on the right side of the plot and the less abundant proteins are on the left side of the graph; from 113 proteins, 39 were up regulated and 74 downregulated (Fig. 1). A total of 94 deregulated proteins have been reviewed by manually making annotations on UniProt, and 50 deregulated proteins were identified with 3 or more unique peptides, providing high confidence in protein identification (Table 2).

### Enrichment analysis of zika positive and zika negative placenta

The top canonical pathways, diseases, and biological functions identified from differentially expressed proteins between Zika positive and Zika negative mothers were determined by IPA (Table 3).

**Fig. 2:**
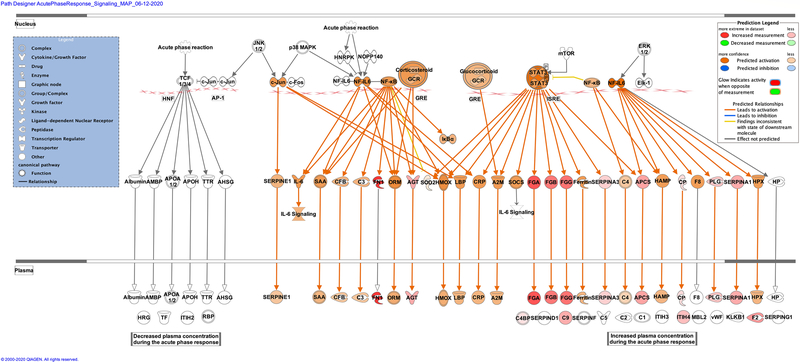
Differentially expressed proteins induced by the Zika virus affect the Nuclear and Plasma Acute Phase Response Signaling Pathway. The cellular region where the differentially abundant proteins were identified for the acute phase response pathway. Proteins in red mean upregulation of the expression. A total of 14 proteins are deregulated in this pathway. These are fibronectin 1 (FN1), fibrinogen alpha chain (FGA), fibrinogen gamma chain (FGG), fibrinogen beta chain (FGB), coagulation factor II (F2), serum amyloid P component (APCS), angiotensinogen (AGT), serpin family A member 1 (SERPINA1), complement C9 (C9), inter-alpha-trypsin inhibitor heavy chain 4 (ITIH4), plasminogen (PLG), serpin family A member 3 (SERPINA3), ceruloplasmin (CP) and complement component 4 binding protein alpha (C4BP). Prediction legend, the intensity of the color red means higher upregulation of the protein. Proteins in the color orange in the diagrams were not detected by our proteomics experiments. The color orange means predicted activation of the protein or interaction leading to activation. The color yellow in the lines of the pathway means inconsistency due to downstream deregulated proteins. The protein gene name is used in the diagrams, to identify proteins look at Supplementary Table 1 gene name column. Data were analyzed through the use of IPA (QIAGEN Inc., https://www.qiagenbioinformatics.com/products/ingenuitypathway-analysis). (Color figure online)

**Fig. 3:**
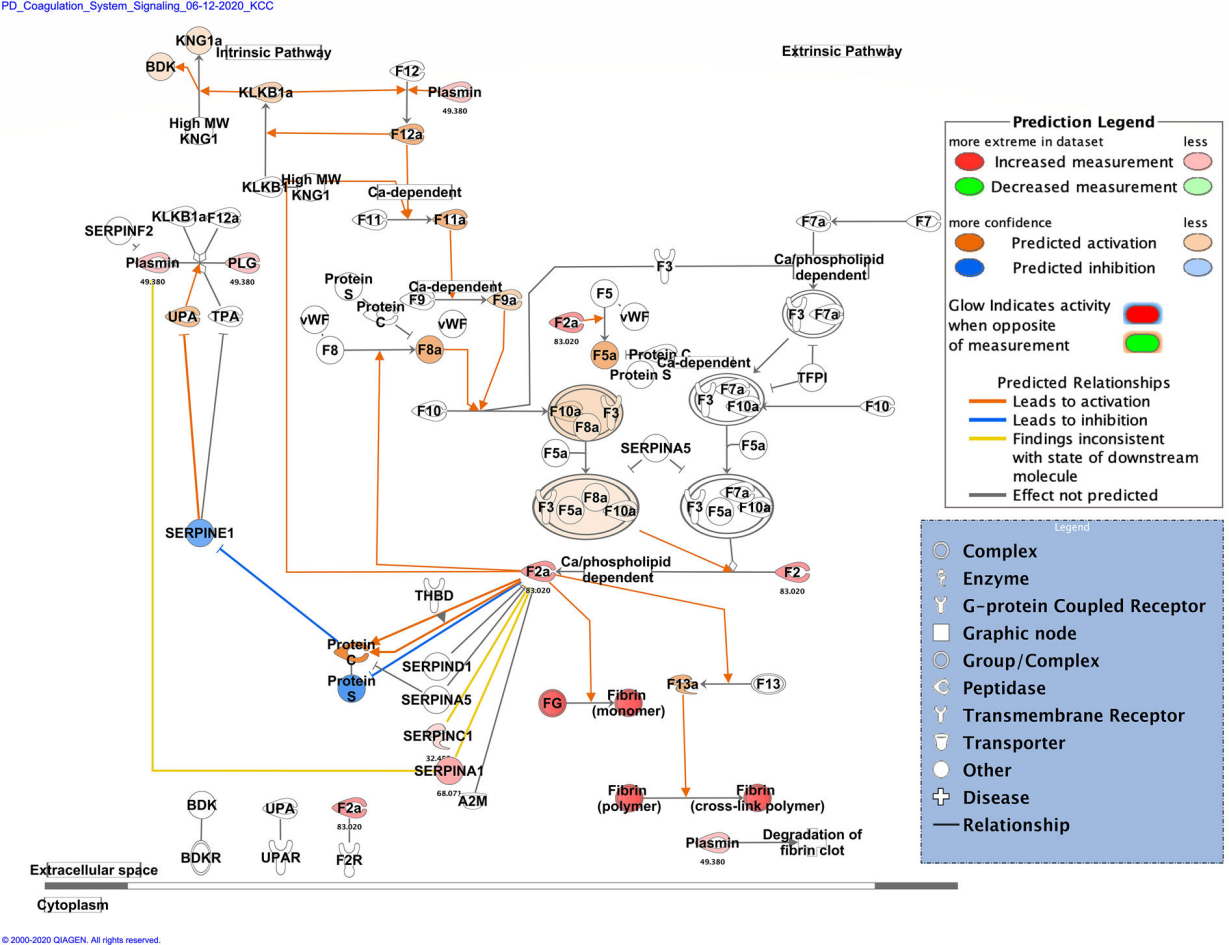
Differentially expressed proteins induced by the Zika virus affect the coagulation system pathway. Proteins in red mean upregulation of the expression. A total of 7 proteins are deregulated in this pathway. These were coagulation factor II (F2 and F2a), fibrinogen (FG and plasmin) [alpha (FGA), gamma (FGG) and beta chains (FGB)], serpin family A member 1 (SERPINA1), serpin family C member 1 (SERPINC1) and plasminogen (PLG and Plasmin). Fold change value is annotated under the proteins. The intensity of the color red means higher upregulation of the protein. Proteins in the color orange or blue depicted in the diagrams were not detected by our proteomics experiments. Prediction legend, the color orange means predicted activation of the protein or interaction leading to activation. The color blue means inhibition of the protein or interaction leading to inhibition. The color yellow in the lines of the pathway means inconsistency due to downstream deregulated proteins. The protein gene name is used in the diagrams, to identify proteins look at Supplementary Table 1 gene name column. Data were analyzed through the use of IPA (QIAGEN Inc., https://www.qiagenbioinformatics.com/products/ingenuitypathway-analysis). (Color figure online)

**Fig. 4:**
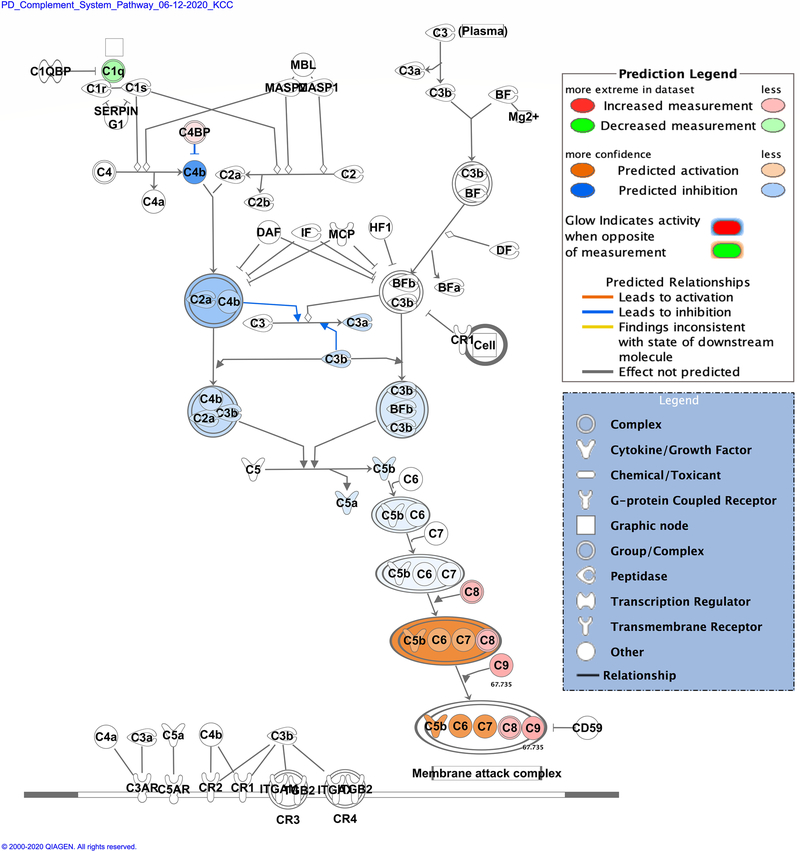
Differentially expressed proteins induced by the Zika virus affect the Complement System pathway. Proteins in red mean upregulation of the expression and proteins in green mean downregulated expression. 4 proteins were identified in this pathway. The upregulated proteins are complement C9 (C9), complement component 4 binding protein alpha (C4BP) and complement C8 gamma chain (C8). The only downregulated protein in this pathway is the complement C1q B chain (C1q). Fold change value is annotated under the proteins. Prediction legend, the intensity of the color red means higher upregulation of the protein. Proteins in the color orange or blue depicted in the diagrams were not detected by our proteomics experiments. The color orange means predicted activation of the protein or interaction leading to activation. The color blue means inhibition of the protein or interaction leading to inhibition. The protein gene name is used in the diagrams, to identify proteins look at Supplementary Table 1 gene name column. Data were analyzed through the use of IPA (QIAGEN Inc., https://www.qiagenbioinformatics.com/products/ingenuitypathway-analysis). (Color figure online)

**Fig. 5:**
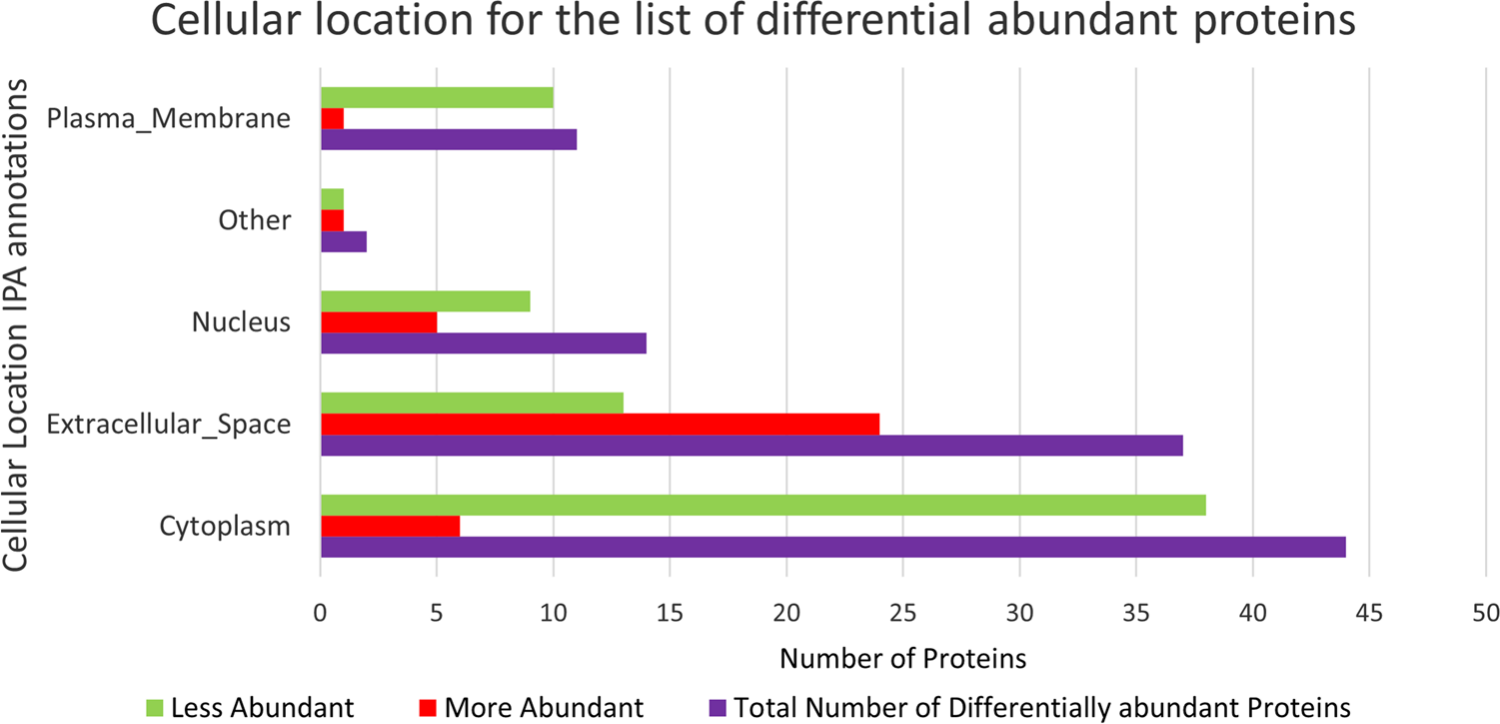
Bar plot of differentially abundant proteins in Zika virus infected placenta that is associated with cellular locations identified in IPA. The plot displays the total number of proteins associated per location, along with the number of proteins that are more and less abundant. Data is stratified as More abundant (red bar), Less abundant (green bar), and a combination of less and more abundant proteins (purple bar). The 113 significantly differentially abundant proteins identified by TMT analysis using the criteria of FC >|2| and a *p* value adjusted with the Benjamini–Hochberg method of ≤ 0.05 are present in this graph. (Color figure online)

Zika infection in the placenta is affecting the inflammatory and coagulation pathways. At least 26% of deregulated proteins are involved in the development and maintenance of homeostasis of the tissue in the placenta. The Nuclear and Plasma Acute Phase Response Signaling Pathway identified 14 proteins (Fig. 2), and the Coagulation system pathway identified 7 proteins. These upregulated proteins were plasminogen, plasmin, fibrinogen, fibrin, coagulation factor II (thrombin), Alpha-1-antitrypsin, antithrombin (Fig. 3). The Extrinsic prothrombin activation pathway (Supplementary Figure 1) with 5 identified upregulated proteins were found activated: fibrinogen, fibrin, coagulation factor II (thrombin), prothrombin, and antithrombin III. This pathway is essential for the activation of the extrinsic coagulation pathway. The Complement System pathway (Fig. 4) identified four proteins: C4BP, C8, and C9 as upregulated and C1q as downregulated while the membrane attack complex is activated, which is common in an acute response against viral infection. We noticed that significant canonical pathways in the extracellular space of the tissue were most affected. Using IPA, we associated the deregulated proteins with several cellular locations (Fig. 5). From deregulated proteins in Zika infected tissue 39% are in the cytoplasm, 33% in the extracellular space, and 13% in the nucleus.

### Validation of proteomics by three-color immunofluorescence

To validate relevant proteins with higher confidence in the enrichment analysis we used IPA. Two-node networks were generated, the first node confirmed the presence of the proteins present in the different canonical pathways analyzed and their interactions: fibrinogen alpha chain, fibrinogen gamma chain, fibrinogen beta chain, and coagulation factor II, thrombin (Supplementary Figure 2). For the second node network, several filters were applied that included: reviewed proteins in UniProt, and the presence of three or more identified unique peptides per protein (Supplementary Figure 3). Based on this analysis, the following proteins were selected for validation: Alpha-1-antitrypsin, a protein with 23 unique peptides identified as upregulated in ZIKV positive placenta with a fold change of 69.07 and a *p* value of 0.0067. Alpha-1-antitrypsin is present in the coagulation system pathway and acute phase response signaling pathway with 9 interactions with other proteins including those from the coagulation system, intrinsic prothrombin activation, acute phase response signaling, and clathrin-mediated endocytosis pathways. The second protein selected for validation was the fibrinogen gamma chain, a protein with 22 unique peptides identified as upregulated in ZIKV positive placenta with a fold change of 114 and a *p* value of 0.0023. The sequence detected in our TMT results is not reviewed in the UniProt database, but the complete protein is reviewed extensively (Farrel 2004; Shabana et al. 2017). This protein is part of fibrin which is composed of fibrinogen alpha, beta, and gamma chains, which are present in acute phase response signaling, coagulation system, and extrinsic prothrombin activation. Fibrinogen Gamma chain has a total of nine interactions with proteins in these pathways. The third protein selected for validation was Fibronectin, a protein with 41 unique peptides identified as upregulated in ZIKV positive placenta with a fold change of 166.9 and a *p* value of 0.0321. This protein is only present in the acute response signaling pathway, but it has 25 interactions with other deregulated proteins found in these results, making it the protein with most interactions associated with Zika infection. Fibronectin interacts with proteins that participate in acute phase response signaling, coagulation system, extrinsic prothrombin activation, clathrin-mediated endocytosis, and complement system pathways.

**Fig. 6:**
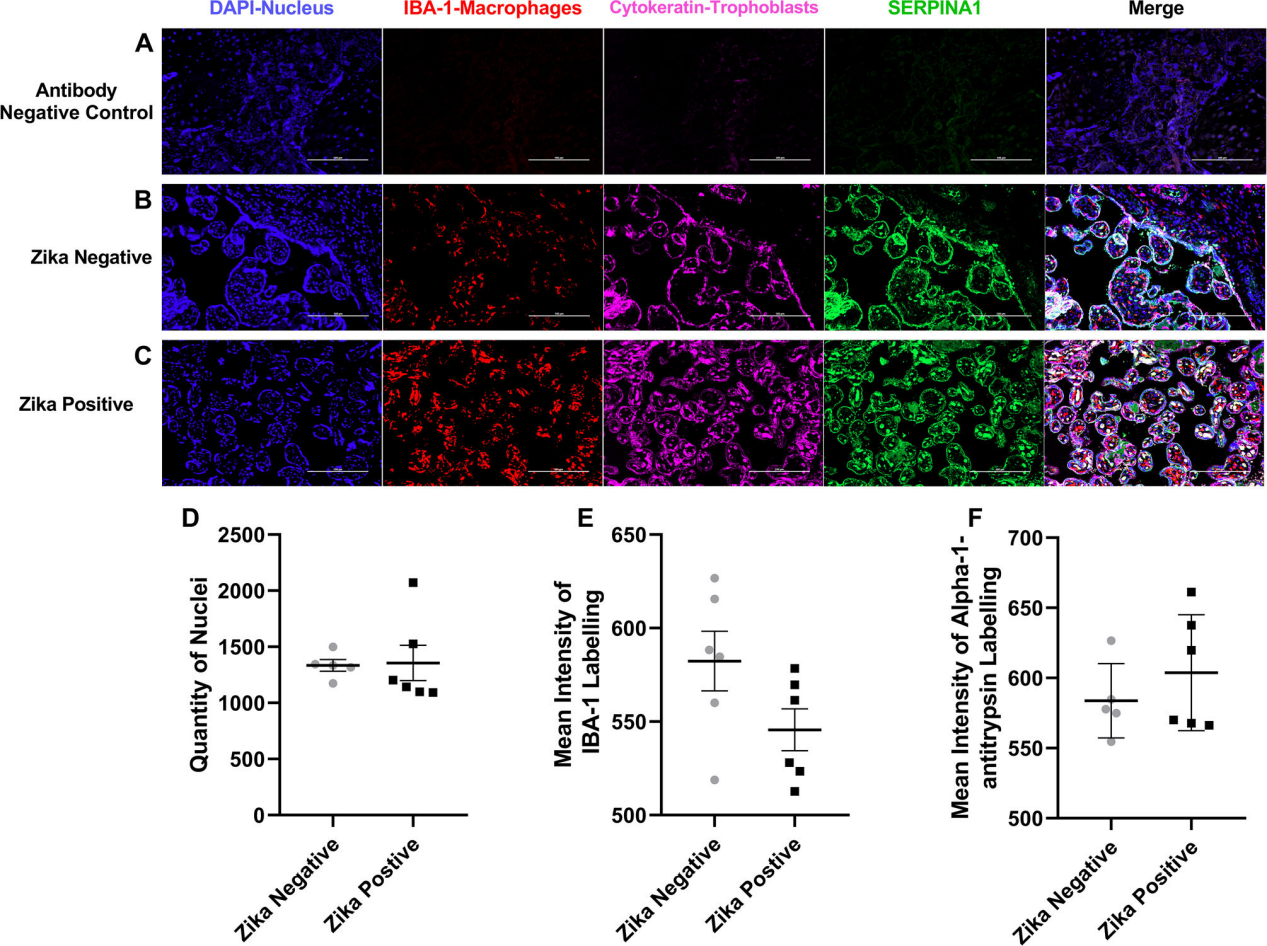
Alpha-1-antitrypsin labeling of Zika positive and Zika negative placentas. **a** Representative fluorescence immunohistochemistry of antibody negative control, **b** ZIKV uninfected placental tissue and **c** ZIKV infected tissue. The tissue is labeled with DAPI for nuclei (Blue), with anti-Iba for placental macrophages or Hofbauer cells (Red), with anti-cytokeratin 8+18 (Pink) and for anti-ERPINA1 protein (Green). Pictures were captured at a magnification of 20X. **d** Differences in quantity of nuclei between ZIKV positive and ZIKV negative samples. There is no statistical difference between the number of nuclei with a *p* value of 0.8993 when the ZIKV negative group was compared to the positive. **e** Differences in Mean intensity between ZIKV positive and ZIKV negative samples of IBA-1 labeling. There is no statistical difference between the mean intensity of the protein IBA-1 with a p-value of 0.0923 between the ZIKV positive and ZIKV negative tissue. **f** Differences in Mean intensity between ZIKV positive and ZIKV negativ e samples of Alpha-1-antitrypsin labeling (SERPINA1). There is no statistical difference between the mean intensity of the protein ATT with a *p* value of 0.3578 when comparing ZIKV positive group to ZIKV negative group. Quantity of nuclei and Mean Intensity was determined using NIS Elements A software object count and ROI Intensity function. This experiment was performed with 12 (*n* = 12) different samples 6 (*n* = 6) positive for ZIKV and 6 (*n* = 6) negative for ZIKV showing no statistical difference between the number of nuclei on the pictures taken for the validation of each protein of interest. There is no significant difference in mean intensity for IBA and Alpha-1-antitrypsin (SERPINA1) between ZIKV positive and ZIKV negative groups. Five pictures were analyzed, and a mean is determined for the number of cells in each sample, ROUT outlier test with *Q* = 1% is performed. A Shapiro–Wilk test with a significance level alpha of 0.05 for *p* value is used to determine distribution because the sample size was too small to perform other normal distribution tests. An unpaired *t* test with Welch’s correction was applied using a 95% confidence as statistically significant (*p ≤ 0.05, **p ≤ 0.01, ***p ≤ 0.001, ****p ≤ 0.0001). (Color figure online)

The first protein tested by immunofluorescence was alpha-antitrypsin-1 (Fig. 6). The background staining was low as shown with the negative controls (Fig. 6a). Alpha-1-antitrypsin can be observed colocalizing with the labeled trophoblast cells and inside the chorionic villi in the stroma under the trophoblast cell layer (Fig. 6b, c). We did not find a statistical difference in the number of cell nuclei (*p* = 0.8993) between ZIKV positive to ZIKV negative samples (Fig. 6d). Alpha-1-antitrypsin appeared to increase in the stroma of ZIKV positive samples while ZIKV Negative samples appeared more concentrated in the trophoblast layer. However, the mean intensity of the IBA-1 (*p* = 0.0923) and alpha-1-antitrypsin (*p* = 0.3578) was not significant between the groups (Fig. 6e, f).

**Fig. 7:**
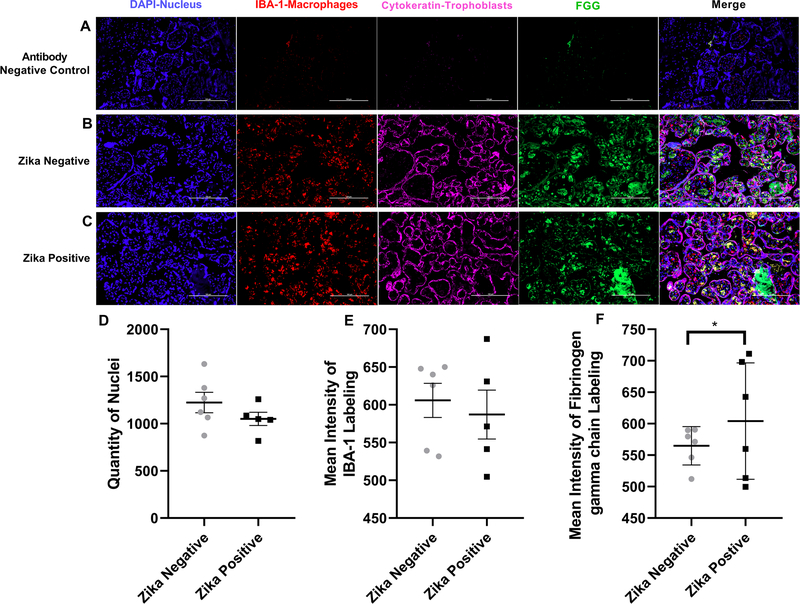
Fibrinogen gamma chain labeling of ZIKV positive and ZIKV negative placentas. **a** Representative Images of Fibrinogen gamma chain labeling. Representative fluorescence immunohistochemistry of antibody negative control, **b** ZIKV uninfected placental tissue and **c** ZIKV infected tissue. The tissue is labeled with DAPI for nuclei (Blue), with anti-Iba for placental macrophages known as Hofbauer cells (Red), with anti-cytokeratin 8+18 (Pink) and for anti-FGG protein (Green). Pictures were captured at a magnification of 20X. **d** Differences in Quantity of Nuclei between ZIKV positive and ZIKV negative samples were not significant (*p* = 0.2156). **e** Mean intensity between ZIKV positive and ZIKV negative samples of IBA labeling show no statistical difference (*p* = 0.6492). **f** Differences in Mean intensity between ZIKV positive and ZIKV negative samples of Fibrinogen gamma chain (FGG) labeling was significant (*p* = 0.0456) showing increased Fibrinogen gamma chain expression in ZIKV positive compared to ZIKV negative group. Quantity of nuclei and Mean Intensity was determined using NIS Elements A software object count and ROI Intensity function. This experiments with 12 (*n* = 12) different samples 6 (*n* = 6) ZIKV positive and 6 (*n* = 6) ZIKV negative show that there is no statistical difference between the number of nuclei on the pictures taken for the validation of each protein of interest. For IBA labeling there is no significant statistical difference between Zika positive group and the Zika negative. There is a significant difference in mean intensity for the Fibrinogen gamma chain labeling Zika positive group and Zika negative. 5 pictures are analyzed, and a mean is determined for the number of cells in each sample, ROUT outlier test with *Q* = 1% is performed. A Shapiro–Wilk test with a significance level alpha of 0.05 for p-value is used to determine distribution because the sample size was too small for other normal distribution tests was performed. An unpaired t-test with Welch’s correction was applied using a 95% confidence as statistically significant (*p ≤ 0.05, **p ≤ 0.01, ***p ≤ 0.001, ****p ≤ 0.0001). (Color figure online)

The second protein studied by immunofluorescence was the fibrinogen gamma chain (Fig. 7). The background staining was low as shown with the negative controls (Fig. 7a). The fibrinogen gamma chain is localized mostly in the stroma of the chorionic villi and less quantity colocalizing with the trophoblast layer surrounding the chorionic villi of Zika negative placenta (Fig. 7b). In Zika positive samples Fibrinogen gamma chain labeling is observed in more areas in the stroma (Fig. 7c). We observed colocalization of fibrinogen gamma chain labeling with IBA-1 labeling in the stroma of the chorionic villi. We did not find a statistical difference (*p* = 0.2156) between the number of cell nuclei detected when compared to ZIKV positive to ZIKV negative samples indicating that both tissues had a similar number of cells (Fig. 7d). IBA-1 intensity was not significantly different between the two groups (*p* = 0.6492) indicating that the number of macrophages was not different between the ZIKV positive and negative groups (Fig. 7e). Deposits of fibrinogen gamma chain protein can be observed inside the chorionic villi on ZIKV negative and ZIKV positive samples but the mean intensity of the fibrinogen gamma chain was higher in ZIKV positive placental tissue when compared to ZIKV negative placenta (*p* = 0.0456; Fig. 7f).

**Fig. 8:**
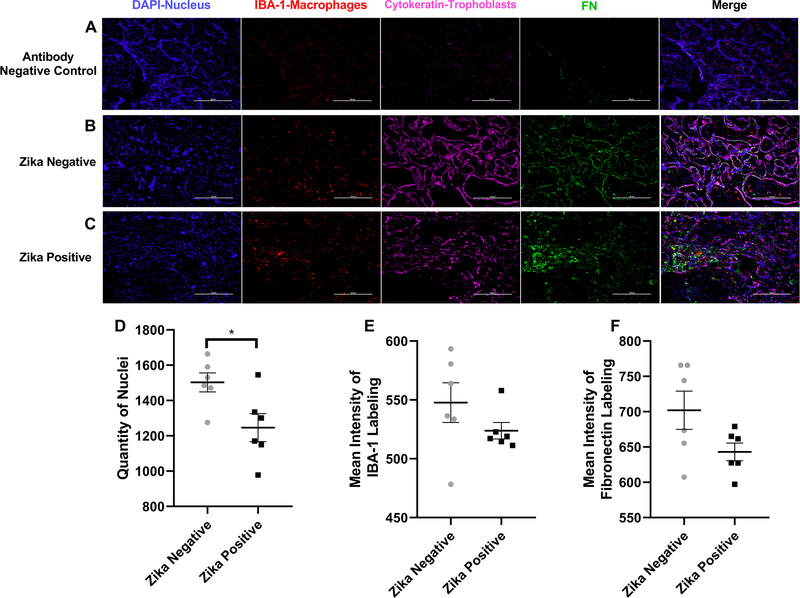
Fibronectin labeling of Zika positive and Zika negative placentas. **a** Representative Images of Fibronectin labeling. Representative fluorescence immunohistochemistry of antibody negative control, **b** ZIKV uninfected placental tissue and **c** ZIKV infected tissue. The tissue is labeled with DAPI for nuclei (Blue), with anti-IBA-1 for placental macrophages known as Hofbauer cells (Red), with anti-cytokeratin 8+18 (Pink) and for anti-FN protein (Green). Pictures were captured at a magnification of 20X. **d** Differences in quantity of nuclei between ZIKV positive and ZIKV negative samples. There is a statistical difference between the number of nuclei with a *p* value of 0.0258 when the ZIKV negative group was compared to the positive. **e** Differences in Mean intensity between ZIKV positive and ZIKV negative samples of IBA-1 labeling were not significant (*p* = 0.2347). **f** Differences in mean intensity between ZIKV positive and ZIKV negative samples of fibronectin (FN1) labeling. There is no statistical difference between the mean intensity of the protein Fibronectin with a *p* value of 0.0877 when comparing ZIKV positive to ZIKV negative group. Quantity of nuclei and Mean Intensity was determined using NIS Elements A software object count and ROI Intensity function. This experiments with 12 (*n* = 12) different samples 6 (*n* = 6) positive for ZIKV and 6 (*n* = 6) negative for ZIKV shows that there is a statistical difference between the number of nuclei on the pictures taken for the validation of each protein of interest between the groups. There is no significant difference in mean intensity and IBA-1 and fibronectin (FN1) between ZIKV positive and ZIKV negative group. Five pictures are analyzed, and a mean is determined for the number of cells in each sample, ROUT outlier test with *Q* = 1% is performed. A Shapiro–Wilk test with a significance level of *p* < 0.05 is used to determine distribution. An unpaired *t* test with Welch’s correction was applied using a 95% confidence as statistically significant (*p ≤ 0.05, **p ≤ 0.01, ***p ≤ 0.001, ****p ≤ 0.0001). (Color figure online)

The third protein studied by immunofluorescence was fibronectin (Fig. 8). The background staining was low as shown with the negative controls (Fig. 8a). Fibronectin labeling in ZIKV negative samples (Fig. 8b) can be observed colocalizing with the cytokeratin labeling identifying the trophoblast, in contrast to ZIKV positive samples (Fig. 8c) where the fibronectin labeling can be observed in the stroma of the chorionic villi. In the staining of fibronectin labeled tissue, there was a statistical difference between the number of cell nuclei between ZIKV positive and ZIKV negative samples (*p* = 0.0258), showing a higher quantity of nuclei in ZIKV negative samples (Fig. 8d). Fibronectin is not colocalizing with the IBA-1 labeling like the fibrinogen gamma chain. The mean intensity of the labeled proteins IBA-1 and fibronectin were not statistically significantly different between the ZIKV positive and ZIKV negative samples with a *p* value 0.2347 and of 0.0877 respectively (Fig. 8e, f).

## Discussion

In this study, we have applied a quantitative proteomics approach to understand the changes in the placenta proteome after ZIKV infection and to identify proteins and pathways affected by the virus that facilitate entry across the barrier formed in the placenta and gain access to the developing fetus. We used ex-vivo tissue of placenta from ZIKV infected mothers collected during delivery and formalin fixed, which gives us a close model to the in-vivo window at the moment of the sample collection. To our knowledge, we are the first group that applied TMT 10-plex a robust proteomics method technique to compare the full proteome of ZIKV infected placental tissue from Puerto Rican women. Previously published research studies using proteomics techniques focused on, how the virus crosses the blood–brain barrier, the neurological effects of the ZIKV infection, and the ZIKV Congenital Syndrome defects that are present at birth or develop thereafter. It has been five years since the first ZIKV pandemic started and there is still a knowledge gap of how ZIKV crosses the placental barrier.

Following mass spectrometry and IPA, we determined that the coagulation and the acute phase response signaling pathways have the highest quantity of significantly more abundant proteins in ZIKV infected placental tissue. Fibrinogen (FG), the precursor of fibrin, has a higher fold change in comparison to the other proteins of the pathway. The upregulation of FG in the placental tissue suggests that ZIKV infection is promoting the coagulation of placental tissue. The coagulation pathway happens exclusively in the extracellular space of tissues. The proteins in this pathway are commonly found circulating in the blood. Although coagulation is commonly associated with an injury, the proteins involved have functions in the innate immune system and tissue remodeling as well, thus the antiviral innate immune response to ZIKV might be inducing these pathways. Since the proteins fibrinogen-α (FGA), fibrinogen-β (FGB), and fibrinogen-γ (FGG) are overexpressed, and these are precursors of fibrin, a major component of blood clots, these data indicate that the coagulation cascade is being activated in the placental tissue upon ZIKV infection. After validation of the proteins alpha-1 antitrypsin, fibrinogen gamma chain, fibronectin by IHC we found that in non-infected placentas these three proteins are mostly located in the trophoblast layer or inside of the chorionic villi. Our observation of alpha-1 antitrypsin, fibrinogen gamma chain, fibronectin being localized in the stroma of the tissue suggests that ZIKV infection of the placenta is also activating a restructuration of ECM by activating these proteins. Colocalization of fibrinogen gamma chain with the labeled macrophages and fibronectin location in the stroma in ZIKV infection further supports our proteomics results of the coagulation pathway being active. The fibrinogen gamma chain in its fibrin form is deposited to the site affected as a temporary measurement to stop the damage from spreading through the tissue until macrophages and fibroblast can remodel the area. Fibroblasts secrete fibronectin which will be assembled into an insoluble matrix. In our results, we did not find a significant difference between IBA-1 expression in Zika negative tissue and Zika positive tissue indicating the lack of macrophage hyperplasia in the placenta tissues studied. Our results differ from other studies from a placenta of 11-week gestation of a fetus that was spontaneously aborted and was characterized by increased macrophage hyperplasia (Rosenburg et al. 2017); and also from Miranda et al. (2019) study in which infants were born prematurely with fetal defects. Differences from our study work and Rosenburg and Miranda studies include the stage of the placenta and the infant outcomes. Future proteomics studies will compare the placenta from an increased number of ZIKV positive and negative infants.

However, the proteomics results presented here, give us insight into what could be happening to permit ZIKV crossing of the placental barrier. The activation of the acute phase response and the coagulation pathways could be causing a restructuration of the placental tissue which benefits ZIKV spread across the placenta, but with the up-regulated proteins common for both pathways and the colocalization of the macrophages with fibrinogen gamma chain could suggest that ZIKV is taking advantage of the anti-inflammatory properties of fibrin, the insoluble product of fibrinogen in macrophages (Hsieh et al. 2017). Due to limitations in commercial antibody availability, we could only identify fibrinogen in its soluble form as our antibody targeted fibrinogen gamma chain only. An antibody that targets fibrin, the insoluble product of fibrinogen was not found for testing colocalization in placental macrophages. Further pathway inhibition and protein identification studies could confirm these results. However, our results demonstrate that these ECM proteins are affected by ZIKV infection and could be potential targets of therapy against vertical transmission and CZV syndrome.

## Supplementary Material

Supplementary Figure

Supplementary Table

## Figures and Tables

**Table 1:** TMT placental sample labeling for comparison of differential expression between five Zika (+) and four Zika (−) control groups

TMT labels		
TMT-126	Zika + sample 20	126.12773
TMT-127N	Zika − sample 22	127.12476
TMT-127C	Zika − sample 24	127.13108
TMT-128N	Zika − sample 32	128.12812
TMT-128C	Zika + sample 1	128.13444
TMT-129N	Zika + sample 2	129.13147
TMT-129C	Zika + sample 3	129.13779
TMT-130N	Zika + sample 4	130.13483
TMT-130C	Zika − sample 0	130.14115
TMT-131	N/A	N/A

**Table 2:** Differentially abundant proteins were identified between Zika (+) versus Zika (−) groups. (Color Table online)

**Comparison**	**Up regulated**	**Down regulated**	**Total Differentially Abundant proteins**
**Zika (+) vs Zika (−)**	39	74	113
**Review**			**Total Proteins Reviewed and Unreviewed**
**Reviewed Proteins**	32	62	94
**Unreviewed Proteins**	7	12	19
**Unique Peptides**			**Total Proteins with ≥ 3 Unique Peptides**
≥ 3	22	28	50

Proteins were filtered by the UniProt database (The UniProt Consortium 2021) by reviewed and unreviewed criteria and filtered by ≥ 3 unique peptides criteria. Total protein with an FC >|2| and a *p* value adjusted with the Benjamini–Hochberg method of ≤ 0.05. Fold change comparisons for this and other tables and figures: Significant Up-regulated Proteins = Red; Significant Down-regulated Proteins = Green

**Table 3:** Top canonical pathways affected by ZIKV infection in the placenta

Top canonical pathways	

Name	*P* value

Acute phase response signaling	1.48 × 10^–13^
Coagulation system	2.66 × 10^–10^
FXR/RXR activation	8.91 × 10^–09^
Extrinsic prothrombin activation pathway	9.13 × 10^–09^
LCR/RXR activation	1.13 × 10^–09^
Top diseases and bio functions	

Name	#Molecules

Diseases and disorders	
Inflammatory response	49
Neurological disease	42
Organismal injury and abnormalities	102
Metabolic disease	43
Psychological disorders	36
Molecular and cellular functions	
Cellular compromise	28
Protein synthesis	40
Cellular movement	41
Cell-to-cell signaling and interaction	34
Protein degradation	20

Physiological system development and function	

Tissue development	35
Hematological system development and function	39
Immune cell trafficking	25
Organismal functions	12
Renal and urological system development and function	14

The top 5 pathways and diseases or biological functions with the highest number of proteins that have a significant change in abundance are described here. The name of the disease or function is in the left column and the number of proteins identified is in the right column identified as # molecules. This table includes reviewed and unreviewed proteins in UniProt

## Data Availability

The mass spectrometry proteomics data have been deposited to the ProteomeXchange (Deutsch et al. 2020) Consortium via the PRIDE (Perez-Riverol et al. 2019) partner repository with the dataset identifier PXD023964.
