# Supplementary material for: Zika virus infection of the placenta alters extracellular matrix proteome
Source: J Mol Histol. Author manuscript; Available in PMC 2022 May 19. (PMC8760362; doi:10.1007/s10735-021-09994-w)
Supplement: Supplementary file 1 [file NIHMS1739740-supplement-Supplementary_Figure.docx]

**Supplementary Figure 1. Extrinsic prothrombin activation pathway** **affected by Zika virus infection**. Proteins in red are upregulated. Five proteins were identified in this pathway as upregulated (Red): coagulation factor II (F2, prothrombin and thrombin), fibrinogen/fibrin (Alpha, beta and gamma chain), and serpin family C member 1 (ATIII). Fold change value is annotated under the proteins. Prediction legend, the intensity of the color red means higher upregulation of the protein. Proteins in the color orange or blue depicted in the diagrams were not detected by our proteomics experiments. The color orange means predicted activation of the protein or interaction leading to activation. The color blue means inhibition of the protein or interaction leading to inhibition. The protein gene name is used in the diagrams, to identify proteins look at Supplementary Table 1 gene name column.  Data were analyzed through the use of IPA (QIAGEN Inc., https://www.qiagenbioinformatics.com/products/ingenuitypathway-analysis).

**Supplementary Figure 2. Common Proteins and canonical pathways affected by Zika virus infection.** Common proteins identified in the four pathways by` IPA. 1) Acute phase response signaling (14 proteins were identified in this pathway). 2) Coagulation system pathway (7 proteins were identified in this pathway). 3) Extrinsic prothrombin activation pathway (5 proteins were identified in this pathway). 4) Complement System Pathway (4 proteins were identified in this pathway). Fold change value is annotated under the proteins.  A complete line between proteins means a direct interaction a dotted line indicates indirect interaction. The protein gene name is used in the diagrams, to identify proteins look at Supplementary Table 1 gene name column.  Data were analyzed through the use of IPA (QIAGEN Inc.,https://www.qiagenbioinformatics.com/products/ingenuitypathway-analysis).

**Supplementary Figure 3. Interaction network of proteins affected by Zika virus.** The interaction network of proteins affected by the Zika virus was reviewed in UniProt with ≥ 3 unique peptides per protein. This interaction network is composed of the affected proteins and the pathways that were more impacted in our results. Fold change value is annotated under the proteins.  A complete line between proteins means a direct interaction a dotted line indicates indirect interaction. Letter Key: A (Activation), E (Expression), I (Inhibition), L (Proteolysis), LO (Localization), M (Biochemical Modification), P (Phosphorylation/Dephosphorylation), PD (Protein-DNA binding), PP (Protein-Protein binding), RB (Regulation of binding) and T (Transcription). The protein gene name is used in the diagrams, to identify proteins look at **Supplementary Table 1** gene name column. Data were analyzed through the use of IPA (QIAGEN Inc., https://www.qiagenbioinformatics.com/products/ingenuitypathway-analysis)
