# Supplementary material for: Zika virus infection of the placenta alters extracellular matrix proteome
Source: J Mol Histol. Author manuscript; Available in PMC 2022 May 19. (PMC8760362; doi:10.1007/s10735-021-09994-w)
Supplement: Supplementary file 2 [file NIHMS1739740-supplement-Supplementary_Table.docx]

**Supplementary Table 1.** Upregulated proteins have a fold change colored in red and downregulated proteins in green. The table includes Uniprot Accession number, gene name, protein name, if the protein has been reviewed, number of amino acids, molecular weight in Kilodaltons, peptides, and the number of unique peptides

| **Supplementary Table 1. Differentially expressed proteins in ZIKV positive and ZIKV negative placenta** |  |  |  |  |  |  |  |  |  |
| --- | --- | --- | --- | --- | --- | --- | --- | --- | --- |
|  |  |  |  |  |  |  |  |  |  |
| **Uniprot Accession** | **Gene name** | **Protein name** | **Reviewed** | **# AAs** | **MW [kDa]** | **Peptides** | **Unique Peptides** | **Fold- change** | **p-value** |
|  |  |  |  |  |  |  |  |  |  |
| **P02751-15** | FN1 FN | Fibronectin | Yes | 2477 | 272.2 | 41 | 41 | 166.9 | 0.0321 |
| **P13727** | PRG2 MBP | Bone marrow proteoglycan | Yes | 222 | 25.2 | 3 | 3 | 143.4 | 0.0449 |
| **P02671** | FGA | Fibrinogen alpha chain | Yes | 866 | 94.9 | 22 | 22 | 138.7 | 0.004 |
| **C9JC84** | FGG | Fibrinogen gamma chain | No | 461 | 52.3 | 15 | 15 | 114.2 | 0.0023 |
| **P02675** | FGB | Fibrinogen beta chain | Yes | 491 | 55.9 | 20 | 20 | 109.7 | 0.0049 |
| **P19801-2** | AOC1 ABP1 DAO1 | Amiloride-sensitive amine oxidase | Yes | 770 | 87.2 | 7 | 7 | 105.9 | 0.0242 |
| **P52943-2** | CRIP2 CRP2 | Cysteine-rich protein 2 | Yes | 282 | 30.2 | 1 | 1 | 100.6 | 0.0479 |
| **P04792** | HSPB1 HSP27 HSP28 | Heat shock protein beta-1 | Yes | 205 | 22.8 | 10 | 10 | 94.45 | 0.0321 |
| **P10909-2** | CLU APOJ CLI KUB1 AAG4 | Clusterin | Yes | 501 | 57.8 | 4 | 4 | 92.02 | 0.0204 |
| **A0A3B3IQ51** | CFHR2 | Complement factor H-related protein 2 | No | 355 | 39.4 | 1 | 1 | 85.17 | 0.0067 |
| **P00734** | F2 | Prothrombin | Yes | 622 | 70 | 2 | 2 | 83.02 | 0.0404 |
| **A0A286YEY1** | IGHA1 | Immunoglobulin heavy constant alpha 1 | No | 398 | 42.8 | 7 | 7 | 79.13 | 0.0368 |
| **P04004** | VTN | Vitronectin | Yes | 478 | 54.3 | 7 | 7 | 77.35 | 0.0014 |
| **P02743** | APCS PTX2 | Serum amyloid P-component | Yes | 223 | 25.4 | 2 | 2 | 75.26 | 0.0206 |
| **Q92994** | BRF1 BRF GTF3B TAF3B2 TAF3C | Transcription factor IIIB 90 kDa subunit | Yes | 677 | 73.8 | 1 | 1 | 74.52 | 0.0023 |
| **P02511** | CRYAB CRYA2 HSPB5 | Alpha-crystallin B chain | Yes | 175 | 20.1 | 3 | 3 | 71.16 | 0.0118 |
| **P23142** | FBLN1 PP213 | Fibulin-1 | Yes | 721 | 78.3 | 6 | 4 | 70.22 | 0.0001 |
| **P01019** | AGT SERPINA8 | Angiotensinogen | Yes | 485 | 53.1 | 9 | 9 | 69.06 | 0.0023 |
| **P01009** | SERPINA1 AAT PI PRO0684 PRO2209 | Alpha-1-antitrypsin | Yes | 418 | 46.7 | 23 | 23 | 68.07 | 0.0067 |
| **P02748** | C9 | Complement component C9 | Yes | 559 | 63.1 | 3 | 3 | 67.74 | 0.0109 |
| **P05204** | HMGN2 HMG17 | Non-histone chromosomal protein HMG-17 | Yes | 90 | 9.4 | 2 | 2 | 67.58 | 0.0049 |
| **P21980** | TGM2 | Protein-glutamine gamma-glutamyltransferase 2 | Yes | 687 | 77.3 | 12 | 12 | 56.97 | 0.0263 |
| **P07360** | C8G | Complement component C8 gamma chain | Yes | 202 | 22.3 | 2 | 2 | 56.48 | 0.0121 |
| **Q9BW30** | TPPP3 CGI-38 | Tubulin polymerization-promoting protein family member 3 | Yes | 176 | 19 | 2 | 2 | 55.65 | 0.0138 |
| **B7ZKJ8** | ITIH4 | ITIH4 protein | No | 935 | 103.8 | 6 | 6 | 53.11 | 0.0023 |
| **K7EPF9** | APOC1 | Apolipoprotein C-I | No | 129 | 14.3 | 1 | 1 | 51.34 | 0.0109 |
| **P00747** | PLG | Plasminogen | Yes | 810 | 90.5 | 7 | 7 | 49.38 | 0.0045 |
| **P01011** | SERPINA3 AACT GIG24 GIG25 | Alpha-1-antichymotrypsin | Yes | 423 | 47.6 | 5 | 5 | 38.21 | 0.004 |
| **P35625** | TIMP3 | Metalloproteinase inhibitor 3 | Yes | 211 | 24.1 | 1 | 1 | 35.5 | 0.0497 |
| **B4E1Z4** |  | cDNA FLJ55673, highly similar to Complement factor B | No | 1266 | 140.9 | 4 | 4 | 33.9 | 0.0069 |
| **P13224-2** | GP1BB | Platelet glycoprotein Ib beta chain | Yes | 411 | 43.1 | 1 | 1 | 33.58 | 0.0204 |
| **O95833** | CLIC3 | Chloride intracellular channel protein 3 | Yes | 236 | 26.6 | 1 | 1 | 33.02 | 0.0403 |
| **P01008** | SERPINC1 AT3 PRO0309 | Antithrombin-III | Yes | 464 | 52.6 | 5 | 5 | 32.45 | 0.04 |
| **L7N2F9** |  | Uncharacterized protein (Fragment) | No | 121 | 13.1 | 1 | 1 | 31.14 | 0.04 |
| **A0A0B4J1V0** | IGHV3-15 | Immunoglobulin heavy variable 3-15 | Yes | 119 | 12.9 | 1 | 1 | 30.31 | 0.0232 |
| **P15428** | HPGD PGDH1 SDR36C1 | 15-hydroxyprostaglandin dehydrogenase | Yes | 266 | 29 | 1 | 1 | 30.26 | 0.0325 |
| **P00450** | CP | Ceruloplasmin | Yes | 1065 | 122.1 | 5 | 5 | 30.17 | 0.0364 |
| **O75368** | SH3BGRL | SH3 domain-binding glutamic acid-rich-like protein | Yes | 114 | 12.8 | 1 | 1 | 26.02 | 0.0454 |
| **P04003** | C4BPA C4BP | C4b-binding protein alpha chain | Yes | 597 | 67 | 1 | 1 | 22.08 | 0.0187 |
| **O15145** | ARPC3 ARC21 | Actin-related protein 2/3 complex subunit 3 | Yes | 178 | 20.5 | 2 | 2 | -15.82 | 0.0289 |
| **P17612** | PRKACA PKACA | cAMP-dependent protein kinase catalytic subunit alpha | Yes | 351 | 40.6 | 2 | 2 | -15.97 | 0.0484 |
| **Q5JWF2** | GNAS GNAS1 | Guanine nucleotide-binding protein G(s) subunit alpha isoforms XLas | Yes | 1037 | 111 | 2 | 1 | -17.14 | 0.0454 |
| **H0YMV8** | RPS27L | 40S ribosomal protein S27 | No | 100 | 11.3 | 2 | 2 | -17.49 | 0.0228 |
| **Q13151** | HNRNPA0 HNRPA0 | Heterogeneous nuclear ribonucleoprotein A0 | Yes | 305 | 30.8 | 2 | 1 | -17.5 | 0.0206 |
| **P38646** | HSPA9 GRP75 HSPA9B mt-HSP70 | Stress-70 protein, mitochondrial | Yes | 679 | 73.6 | 4 | 3 | -17.61 | 0.0463 |
| **M0QYZ2** | AP2S1 | AP complex subunit sigma | No | 158 | 18.9 | 1 | 1 | -17.64 | 0.021 |
| **P78371** | CCT2 99D8.1 CCTB | T-complex protein 1 subunit beta | Yes | 535 | 57.5 | 4 | 4 | -19.13 | 0.0289 |
| **Q14697-2** | GANAB G2AN KIAA0088 | Neutral alpha-glucosidase AB | Yes | 966 | 109.4 | 16 | 16 | -19.38 | 0.0327 |
| **P55268** | LAMB2 LAMS | Laminin subunit beta-2 | Yes | 1798 | 195.9 | 23 | 23 | -19.63 | 0.04 |
| **O75369-8** | FLNB FLN1L FLN3 TABP TAP | Filamin-B | Yes | 2633 | 281.5 | 2 | 1 | -19.71 | 0.0325 |
| **P08133** | ANXA6 ANX6 | Annexin A6 | Yes | 673 | 75.8 | 15 | 15 | -19.87 | 0.0487 |
| **Q14112** | NID2 | Nidogen-2 | Yes | 1375 | 151.2 | 14 | 13 | -20.18 | 0.0264 |
| **Q969V3** | NCLN | Nicalin | Yes | 563 | 62.9 | 1 | 1 | -20.23 | 0.0188 |
| **P23381** | WARS IFI53 WRS | Tryptophan--tRNA ligase, cytoplasmic | Yes | 471 | 53.1 | 6 | 6 | -20.26 | 0.0289 |
| **O60716** | CTNND1 KIAA0384 | Catenin delta-1 | Yes | 968 | 108.1 | 1 | 1 | -20.53 | 0.0249 |
| **G3XAI2** | LAMB1 hCG_17112 | Laminin subunit beta-1 | No | 1810 | 200.3 | 6 | 6 | -20.62 | 0.0497 |
| **P30101** | PDIA3 ERP57 ERP60 GRP58 | Protein disulfide-isomerase A3 | Yes | 505 | 56.7 | 19 | 19 | -21.01 | 0.031 |
| **O75367** | H2AFY MACROH2A1 | Core histone macro-H2A.1 | Yes | 372 | 39.6 | 5 | 5 | -21.59 | 0.0109 |
| **P55786** | NPEPPS PSA | Puromycin-sensitive aminopeptidase | Yes | 919 | 103.2 | 3 | 3 | -21.72 | 0.0109 |
| **Q9H7Z7** | PTGES2 C9orf15 PGES2 | Prostaglandin E synthase 2 | Yes | 377 | 41.9 | 1 | 1 | -21.76 | 0.0206 |
| **Q92896-2** | GLG1 CFR1 ESL1 MG160 | Golgi apparatus protein 1 | Yes | 1203 | 137.1 | 1 | 1 | -22.03 | 0.0109 |
| **P02786** | TFRC | Transferrin receptor protein 1 | Yes | 760 | 84.8 | 6 | 6 | -22.35 | 0.0206 |
| **C9JIF9** | APEH | Acylamino-acid-releasing enzyme | No | 737 | 81.6 | 2 | 2 | -22.39 | 0.0187 |
| **P30048** | PRDX3 AOP1 | Thioredoxin-dependent peroxide reductase, mitochondrial | Yes | 256 | 27.7 | 4 | 4 | -22.41 | 0.0303 |
| **P39059** | COL15A1 | Collagen alpha-1(XV) chain | Yes | 1388 | 141.6 | 13 | 13 | -22.48 | 0.0276 |
| **A8K878** | MANF | Mesencephalic astrocyte-derived neurotrophic factor mRNA) | No | 185 | 21.1 | 1 | 1 | -23 | 0.0373 |
| **Q16853** | AOC3 VAP1 | Membrane primary amine oxidase | Yes | 763 | 84.6 | 1 | 1 | -23.24 | 0.0249 |
| **P02746** | C1QB | Complement C1q subcomponent subunit B | Yes | 253 | 26.7 | 2 | 2 | -23.27 | 0.0118 |
| **A0A3B3ISG8** |  | Uncharacterized protein | No | 567 | 64.2 | 1 | 1 | -23.3 | 0.0262 |
| **A0A2R8YEP4** | BLVRB | Flavin reductase | No | 245 | 26.3 | 4 | 4 | -23.59 | 0.0121 |
| **P30040** | ERP29 C12orf8 ERP28 | Endoplasmic reticulum resident protein 29 | Yes | 261 | 29 | 1 | 1 | -23.66 | 0.0321 |
| **P53396** | ACLY | ATP-citrate synthase | Yes | 1101 | 120.8 | 2 | 2 | -24.06 | 0.0454 |
| **O95782** | AP2A1 ADTAA CLAPA1 | AP-2 complex subunit alpha-1 | Yes | 977 | 107.5 | 4 | 4 | -24.3 | 0.0109 |
| **P26447** | S100A4 CAPL MTS1 | Protein S100-A4 | Yes | 101 | 11.7 | 3 | 3 | -24.57 | 0.0421 |
| **A0A087X0X3** | HNRNPM | Heterogeneous nuclear ribonucleoprotein M | No | 730 | 77.5 | 2 | 2 | -24.65 | 0.0128 |
| **P05089-2** | ARG1 | Arginase-1 | Yes | 330 | 35.6 | 2 | 2 | -25.22 | 0.0342 |
| **Q9Y646** | CPQ LCH1 PGCP | Carboxypeptidase Q | Yes | 472 | 51.9 | 1 | 1 | -25.25 | 0.0281 |
| **P07237** | P4HB ERBA2L PDI PDIA1 PO4DB | Protein disulfide-isomerase | Yes | 508 | 57.1 | 13 | 6 | -25.48 | 0.0172 |
| **P34897** | SHMT2 | Serine hydroxymethyltransferase, mitochondrial | Yes | 504 | 56 | 1 | 1 | -25.56 | 0.0067 |
| **Q16531** | DDB1 XAP1 | DNA damage-binding protein 1 | Yes | 1140 | 126.9 | 4 | 4 | -25.74 | 0.0071 |
| **E7ENQ6** |  | Uncharacterized protein | No | 273 | 30.1 | 1 | 1 | -26.25 | 0.0036 |
| **P56385** | ATP5ME ATP5I ATP5K | ATP synthase subunit e, mitochondrial | Yes | 69 | 7.9 | 1 | 1 | -26.53 | 0.0086 |
| **P21810** | BGN SLRR1A | Biglycan | Yes | 368 | 41.6 | 6 | 6 | -26.59 | 0.0049 |
| **P55899** | FCGRT FCRN | IgG receptor FcRn large subunit p51 | Yes | 365 | 39.7 | 1 | 1 | -26.68 | 0.0397 |
| **Q5TDH0-3** | DDI2 | Protein DDI1 homolog 2 | Yes | 419 | 46.5 | 1 | 1 | -27.41 | 0.0067 |
| **P09661** | SNRPA1 | U2 small nuclear ribonucleoprotein A' | Yes | 255 | 28.4 | 1 | 1 | -27.42 | 0.0118 |
| **Q92743** | HTRA1 HTRA PRSS11 | Serine protease HTRA1 | Yes | 480 | 51.3 | 1 | 1 | -28.43 | 0.0045 |
| **P07738** | BPGM | Bisphosphoglycerate mutase | Yes | 259 | 30 | 2 | 2 | -28.59 | 0.0289 |
| **P08842** | STS ARSC1 | Steryl-sulfatase | Yes | 583 | 65.5 | 5 | 5 | -29.23 | 0.04 |
| **P08134** | RHOC ARH9 ARHC | Rho-related GTP-binding protein RhoC | Yes | 193 | 22 | 2 | 2 | -29.47 | 0.0023 |
| **P49593** | PPM1F KIAA0015 POPX2 | Protein phosphatase 1F (Protein fem-2 homolog) (hFem-2) | Yes | 454 | 49.8 | 1 | 1 | -29.95 | 0.0249 |
| **U3KQK0** | HIST1H2BN hCG_1743059 | Histone H2B | No | 166 | 18.8 | 10 | 2 | -30.25 | 0.019 |
| **P30613** | PKLR PK1 PKL | Pyruvate kinase PKLR | Yes | 574 | 61.8 | 3 | 2 | -30.33 | 0.0373 |
| **P35030** | PRSS3 PRSS4 TRY3 TRY4 | Trypsin-3 | Yes | 304 | 32.5 | 1 | 1 | -30.48 | 0.0484 |
| **P09382** | LGALS1 | Galectin-1 | Yes | 135 | 14.7 | 1 | 1 | -31.01 | 0.0086 |
| **P69905** | HBA1; HBA2 | Hemoglobin subunit alpha | Yes | 142 | 15.2 | 11 | 10 | -31.24 | 0.0264 |
| **P27105** | STOM BND7 EPB72 | Erythrocyte band 7 integral membrane protein | Yes | 288 | 31.7 | 4 | 4 | -31.58 | 0.0023 |
| **P02730** | SLC4A1 AE1 DI EPB3 | Band 3 anion transport protein | Yes | 911 | 101.7 | 7 | 7 | -32.06 | 0.018 |
| **P09622** | DLD GCSL LAD PHE3 | Dihydrolipoyl dehydrogenase, mitochondrial | Yes | 509 | 54.1 | 2 | 2 | -32.56 | 0.0023 |
| **A0A0A0MS15** | IGHV3-49 | Immunoglobulin heavy variable 3-49 | Yes | 119 | 13 | 1 | 1 | -32.9 | 0.0206 |
| **P17900** | GM2A | Ganglioside GM2 activator | Yes | 193 | 20.8 | 1 | 1 | -33.43 | 0.0067 |
| **P46109** | CRKL | Crk-like protein | Yes | 303 | 33.8 | 1 | 1 | -34.18 | 0.0323 |
| **P16403** | HIST1H1C H1F2 | Histone H1.2 | Yes | 213 | 21.4 | 8 | 1 | -34.79 | 0.0049 |
| **Q13228-4** | SELENBP1 SBP | Methanethiol oxidase | Yes | 514 | 56.8 | 9 | 9 | -35.6 | 0.0312 |
| **P02100** | HBE1 HBE | Hemoglobin subunit epsilon | Yes | 147 | 16.2 | 3 | 1 | -36.51 | 0.0414 |
| **P12111** | COL6A3 | Collagen alpha-3(VI) chain | Yes | 3177 | 343.5 | 88 | 67 | -38.11 | 0.0016 |
| **P32119** | PRDX2 NKEFB TDPX1 | Peroxiredoxin-2 (Thioredoxin-dependent peroxide reductase 1) | Yes | 198 | 21.9 | 11 | 10 | -40.31 | 0.0067 |
| **J3KQ32** | OLA1 | Obg-like ATPase 1 | No | 416 | 46.9 | 1 | 1 | -40.62 | 0.0126 |
| **Q5VV89** | MGST3 | Microsomal glutathione S-transferase 3 | No | 166 | 18.4 | 1 | 1 | -40.98 | 0.0085 |
| **Q14722** | KCNAB1 KCNA1B | Voltage-gated potassium channel subunit beta-1 | Yes | 419 | 46.5 | 1 | 1 | -47.58 | 0.0206 |
| **Q6B0K9** | HBM HBAP2 | Hemoglobin subunit mu | Yes | 141 | 15.6 | 3 | 3 | -53.77 | 0.0086 |
| **P10412** | HIST1H1E H1F4 | Histone H1.4 | Yes | 219 | 21.9 | 8 | 1 | -54.44 | 0.0206 |
| **P69892** | HBG2 | Hemoglobin subunit gamma-2 | Yes | 147 | 16.1 | 14 | 2 | -61.55 | 0.0042 |
